# Meta-transcriptomic analysis of companion animal infectomes reveals their diversity and potential roles in animal and human disease

**DOI:** 10.1128/msphere.00439-24

**Published:** 2024-07-16

**Authors:** Wei-Chen Wu, Yuan-Fei Pan, Wu-Di Zhou, Yu-Qi Liao, Min-Wu Peng, Geng-Yan Luo, Gen-Yang Xin, Ya-Ni Peng, Tongqing An, Bo Li, Huanle Luo, Vanessa R. Barrs, Julia A. Beatty, Edward C. Holmes, Wenjing Zhao, Mang Shi, Yuelong Shu

**Affiliations:** 1 National Key Laboratory of Intelligent Tracking and Forecasting for Infectious Diseases, School of Medicine, Shenzhen Campus of Sun Yat-sen University, Sun Yat-sen University, Shenzhen, China; 2 Shenzhen Key Laboratory for Systems Medicine in Inflammatory Diseases, Shenzhen Campus of Sun Yat-sen University, Sun Yat-sen University, Shenzhen, China; 3 Ministry of Education Key Laboratory of Biodiversity Science and Ecological Engineering, School of Life Sciences, Fudan University, Shanghai, China; 4 School of Public Health (Shenzhen), Shenzhen Campus of Sun Yat-sen University, Sun Yat-sen University, Shenzhen, China; 5 State Key Laboratory of Animal Disease Control and Prevention, Harbin Veterinary Research Institute, Chinese Academy of Agricultural Sciences, Harbin, China; 6 Ministry of Education Key Laboratory for Ecosecurity of Southwest China, Yunnan Key Laboratory of Plant Reproductive Adaptation and Evolutionary, Ecology and Centre for Invasion Biology, Institute of Biodiversity, School of Ecology and Environmental Science, Yunnan University, Kunming, China; 7 Department of Veterinary Clinical Sciences, Jockey Club College of Veterinary Medicine and Life Sciences, City University of Hong Kong, Hong Kong SAR, China; 8 Centre for Animal Health and Welfare, City University of Hong Kong, Hong Kong SAR, China; 9 Sydney Institute for Infectious Diseases, School of Medical Sciences, The University of Sydney, Sydney, New South Wales, Australia; 10 Laboratory of Data Discovery for Health Limited, Hong Kong SAR, China; 11 Key Laboratory of Pathogen Infection Prevention and Control (MOE), State Key Laboratory of Respiratory Health and Multimorbidity, National Institute of Pathogen Biology, Chinese Academy of Medical Sciences & Peking Union Medical College, Beijing, China; Nanjing University of Chinese Medicine, Nanjing, China

**Keywords:** zoonoses, veterinary medicine, metagenomics, microbiome, virome

## Abstract

Companion animals such as cats and dogs harbor diverse microbial communities that can potentially impact human health due to close and frequent contact. To better characterize their total infectomes and assess zoonotic risks, we characterized the overall infectomes of companion animals (cats and dogs) and evaluated their potential zoonotic risks. Meta-transcriptomic analyses were performed on 239 samples from cats and dogs collected across China, identifying 24 viral species, 270 bacterial genera, and two fungal genera. Differences in the overall microbiome and infectome composition were compared across different animal species (cats or dogs), sampling sites (rectal or oropharyngeal), and health status (healthy or diseased). Diversity analyses revealed that viral abundance was generally higher in diseased animals compared to healthy ones, while differences in microbial composition were mainly driven by sampling site, followed by animal species and health status. Disease association analyses validated the pathogenicity of known pathogens and suggested potential pathogenic roles of previously undescribed bacteria and newly discovered viruses. Cross-species transmission analyses identified seven pathogens shared between cats and dogs, such as alphacoronavirus 1, which was detected in both oropharyngeal and rectal swabs albeit with differential pathogenicity. Further analyses showed that some viruses, like alphacoronavirus 1, harbored multiple lineages exhibiting distinct pathogenicity, tissue, or host preferences. Ultimately, a systematic evolutionary screening identified 27 potential zoonotic pathogens in this sample set, with far more bacterial than viral species, implying potential health threats to humans. Overall, our meta-transcriptomic analysis reveals a landscape of actively transcribing microorganisms in major companion animals, highlighting key pathogens, those with the potential for cross-species transmission, and possible zoonotic threats.

**IMPORTANCE:**

This study provides a comprehensive characterization of the entire community of infectious microbes (viruses, bacteria, and fungi) in companion animals like cats and dogs, termed the “infectome.” By analyzing hundreds of samples from across China, the researchers identified numerous known and novel pathogens, including 27 potential zoonotic agents that could pose health risks to both animals and humans. Notably, some of these zoonotic pathogens were detected even in apparently healthy pets, highlighting the importance of surveillance. The study also revealed key microbial factors associated with respiratory and gastrointestinal diseases in pets, as well as potential cross-species transmission events between cats and dogs. Overall, this work sheds light on the complex microbial landscapes of companion animals and their potential impacts on animal and human health, underscoring the need for monitoring and management of these infectious agents.

## INTRODUCTION

Cats and dogs are the most common companion animal species. Close contact between pet dogs and cats and their owners raises the possibility of microbial “sharing,” the impacts of which are unclear. On one hand, exposure to companion animals at an early age may provide immune benefits to human health (1). However, living with companion animals might increase the risk of bidirectional transmission of some infectious diseases. Indeed, cats and dogs have been identified as potential sources of zoonoses of varying severity in humans, including viral pathogens (such as rabies virus and norovirus) (2, 3), bacteria (such as *Campylobacter* and *Salmonella*) (4, 5), fungi (such as dermatophytes) (6), as well as parasites (such as *Toxoplasma gondii*) (7), causing human illnesses ranging from mild rashes to severe nerve damage (2). Additionally, companion animals may become infected with some human respiratory viruses, such as influenza viruses (8), as well as multidrug-resistant bacteria, such as extended-spectrum beta-lactamase-resistant *Escherichia coli* and *Clostridium difficile* (9, 10), posing potential risks to human and animal health.

Recently, total RNA sequencing (i.e., meta-transcriptomics) has been used to simultaneously characterize the total “infectome” (i.e., all microbial pathogens within a sample, including bacteria, fungi, and DNA/RNA viruses) of humans (11, 12), wildlife animals (13, 14), livestock species (15), and arthropod vectors (16). Not only does this method identify microbial taxa at the most precise taxonomic level possible, but it also enables accurate estimation of microbial abundance levels by measuring actively transcribing RNA molecules (12, 15). This also facilitates analysis of the interactions between pathogens and microbiota within a diseased system, as demonstrated by the characterization of microbial dysbiosis following SARS-CoV-2 infection in humans (11, 17).

Despite numerous metagenomic studies on cats and dogs, most previous studies have focused on characterizing the commensal bacterial microbiome (18, 19) or discovering novel pathogens (20–22). In contrast, little is known about the entire infectome of companion animals. Herein, we used a meta-transcriptomics approach to perform in-depth comparisons of microbial diversity across different sampling sites (rectal/oropharyngeal swabs), species (dogs/cats), and health status (healthy/diseased), in doing so identifying microbial taxa associated with respiratory and gastrointestinal diseases. In addition, we further characterized the extent and pattern of cross-species microbial transmission among species, and assessed the risk that the microorganisms present in companion animals might pose to animal and human health.

## RESULTS

### Study design

**Fig 1:**
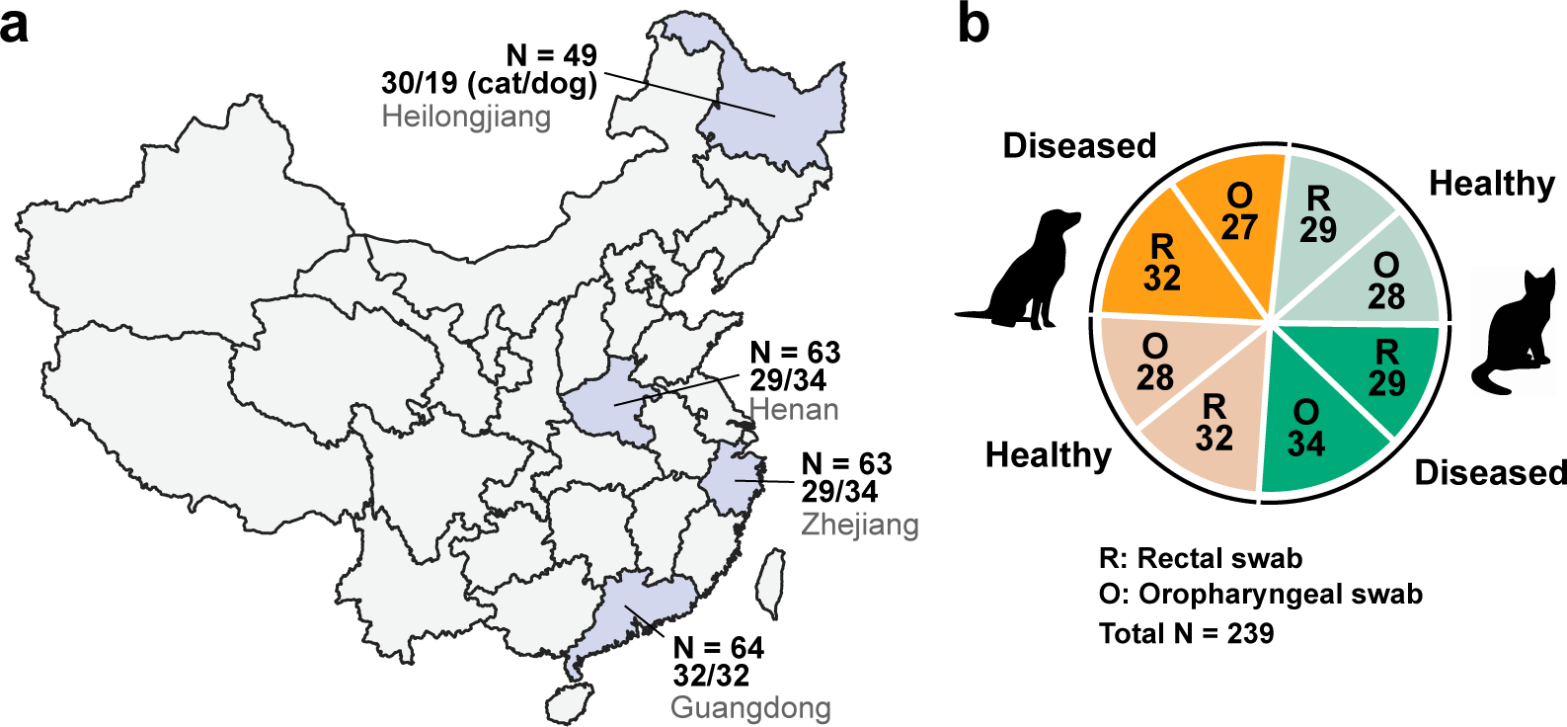
Sample overview. (a) Geographical distribution within China of the companion animal samples collected and the sample size at each location. Open-access map data were obtained at https://doi.org/10.5281/zenodo.4167299. (b) Sample types and corresponding sample sizes.

We sampled a total of 1,130 oropharyngeal and/or rectal swab samples from 571 companion animals—cats and dogs—across four provinces in China. Based on these samples, we set up a balanced experimental design for meta-transcriptomics analyses. This comprised 239 samples from diseased and healthy animals (122:117), cats and dogs (120:119), and oropharyngeal and rectal swabs (117:122) (Fig. 1). The diseased group included animals showing either respiratory (e.g., sneezing, coughing, and nasal discharge) or gastrointestinal signs (e.g., diarrhea and vomiting) (61:61). Within the diseased group, rectal swabs were solely collected from animals with gastrointestinal signs, while oropharyngeal swabs were solely obtained from those with respiratory symptoms. Thus, comparisons between sampling sites in diseased animals in the following analyses implied a comparison between animals showing respiratory and gastrointestinal signs. For each combination (i.e., health status, animal species, and sampling sites), 27–34 individuals were used as replicates, the geographic distributions of which were balanced across the four Chinese provinces in which sampling took place (Fig. 1; Table S1).

### Characterization of total infectome

**Fig 2:**
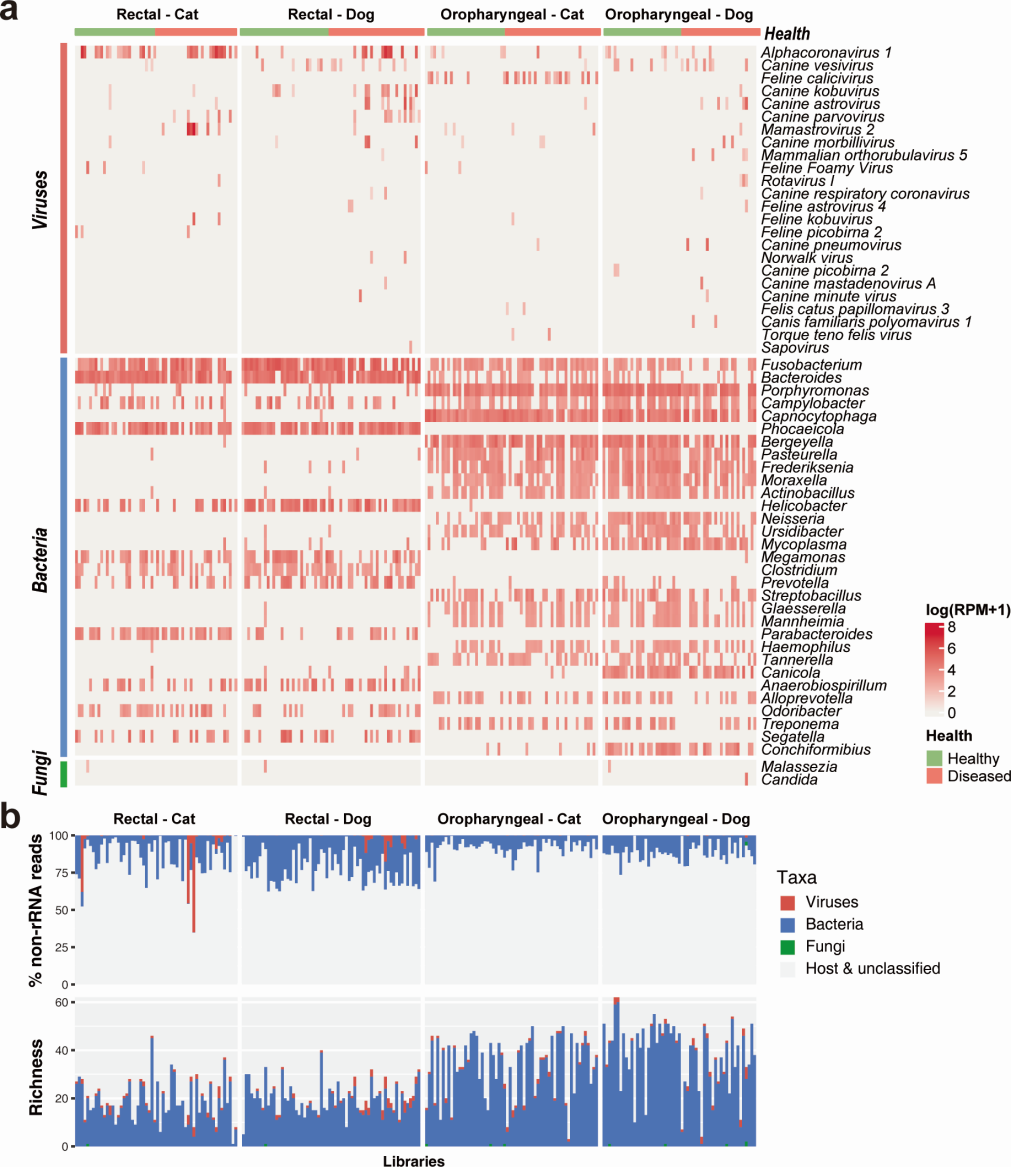
Overview of the infectomes of cats and dogs. (a) Heatmaps illustrating the microbial abundance in each individual sample. The abundance of viruses is quantified at the species level, while bacterial and fungal abundance are quantified at a genus-level resolution. For clarity, bacterial genera with <40 positive samples were omitted from the visual representation. (b) The abundance and diversity of viruses, bacteria and fungi in each library. The diversity of viruses was quantified by species richness (i.e., the number of viral species per sample), while the diversity of bacteria and fungi was quantified by the number of genera.

Our meta-transcriptomic analysis of the total infectome within each sample revealed 24 known mammal-associated viral species, 270 bacterial genera, and two fungal genera (Fig. 2). The virus species identified here comprised 17 RNA viruses, six DNA viruses and one exogenous retrovirus species, in total representing 19 viral genera and 14 families (Fig. S1 and S2). Many of these are well-known pathogens in domestic animals, such as *alphacoronavirus 1*, *feline calicivirus*, *canine parvovirus*, *canine morbillivirus*, and *Norwalk virus*. We also found viruses of uncertain pathogenic potential that were present at high prevalence in our samples, including *canine vesivirus* (*N* = 27 animals), *canine kobuvirus* (*N* = 16), *canine astrovirus* (*N* = 14), and *mamastrovirus 2* (*N* = 13). Generally, an average of 1.61 ± 1.04 (mean ± s.d.) and up to five species of mammal-associated viruses were identified in each animal, with significantly more in diseased than healthy animals (1.87 ± 1.19 in diseased animals, 1.24 ± 0.58 in healthy animals, Wilcoxon test *P* = 0.0006; Fig. S3).

The bacterial genera identified here largely comprised the normal flora of cats and dogs, including those at very high prevalence and abundance (i.e., up to 67% of total non-rRNA reads), such as *Fusobacterium*, *Bacteroides*, *Phocaeicola*, and *Helicobacter* in the rectal swab samples, and *Porphyromonas*, *Capnocytophaga*, and *Bergeyella* in the oropharyngeal swabs (Fig. S4). The dominant bacterial genera differed between rectal and oropharyngeal swabs, but were similar between cats and dogs (Fig. S4). Of note, we identified 23 bacterial species that can infect humans, including *Clostridioides difficile*, *Campylobacter jejuni*, *Moraxella catarrhalis, Capnocytophaga cynodegmi*, and *Helicobacter canis* (Fig. S5; Table S2).

Two fungal species were identified in this study*—Candida albicans* was identified in the oropharyngeal swab sample of a single dog with respiratory symptoms, while *Malassezia globosa* was detected in three healthy animals (two dogs and one cat). Importantly, both species are associated with opportunistic infections of the human skin, respiratory tract, and genitourinary tract, suggesting zoonotic potential.

Overall, based on meta-transcriptomic sequencing, the total infectome accounted for a median of 11.8% (range: 0.02%–65.22%) of total non-rRNA reads (Fig. 2b), many of which belonged to commensal bacteria (median: 11.3% of non-rRNA reads; range: 0.01%–37.66%). In comparison, the mammal-associated viruses were much less abundant (median 0.016% of non-rRNA reads, range 4.8 × 10^−5^% to 65.22%), although in five of the samples, the number of viral reads exceeds 10% of the total non-rRNA reads.

### Comparisons of microbial composition and diversity

**Fig 3:**
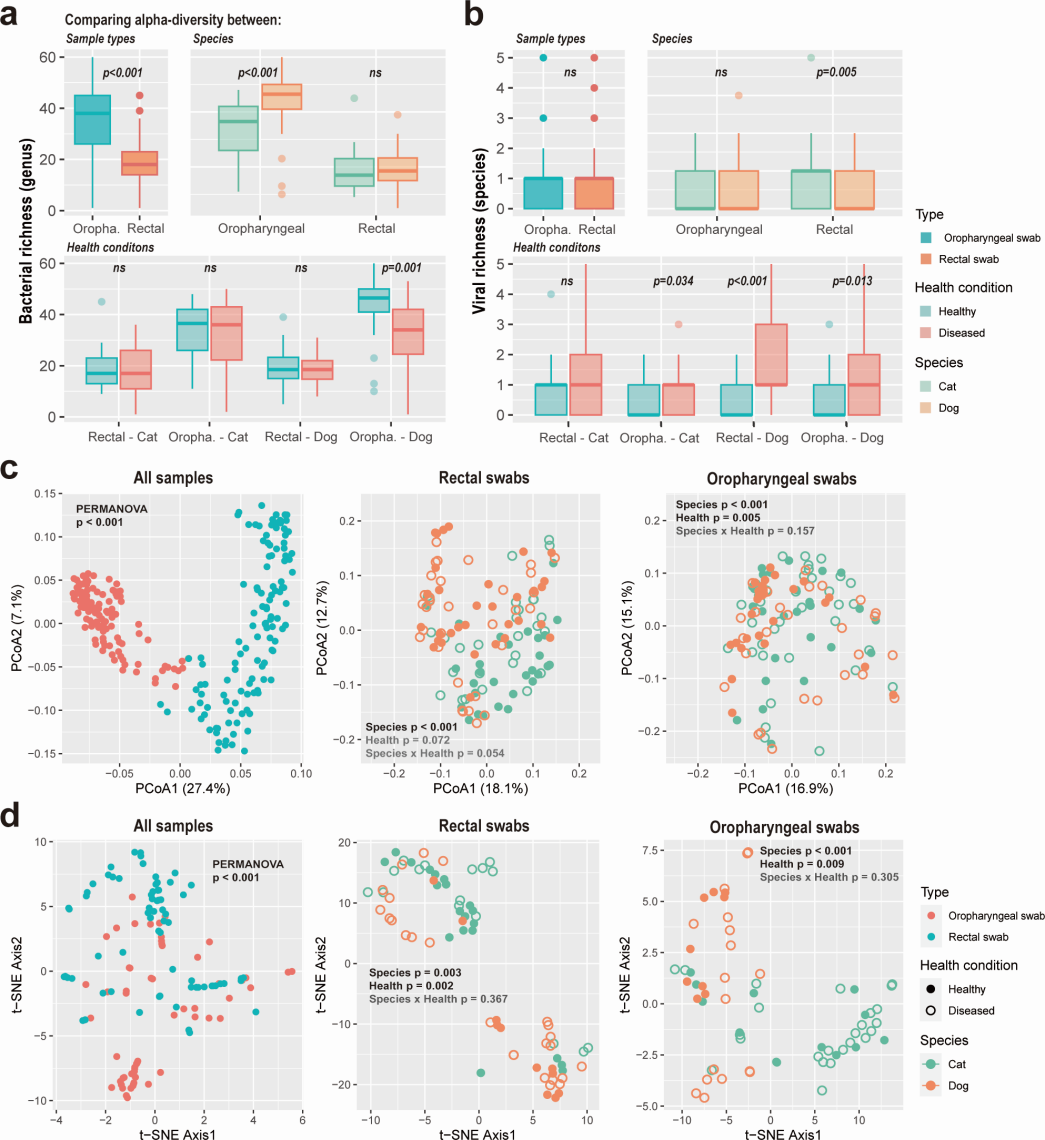
Comparisons of alpha and beta diversity among species, sample types, and health conditions. (a, b) Comparison of bacterial richness (a; number of bacterial genera per sample) and viral richness (b; number of viral species per sample) among sample types (anal/throat swabs), species (cats/dogs), and health conditions (healthy/diseased). (c, d) Comparison of bacterial genera compositions (c) and viral species compositions (d) among samples. *P* values from PERMANOVA tests are shown at the top. For all samples, we only tested the effect of sample type. For specific sample types, we tested the effect of species, health condition, and their interaction. Significant results are shown in bold black fonts.

We compared overall microbial diversity across different sampling sites (oropharyngeal/rectal swabs), animal species (dogs/cats), and health status (healthy/diseased) (Fig. 3a; Fig. S3). The effect of sample location (i.e., provinces) was not considered in these analyses as we found its effect on both viral and bacterial compositions to be insignificant [permutational multivariate analysis of variance (PERMANOVA) tests, *P* ≥ 0.05]. This allowed us to treat samples from the four provinces as independent replications. Oropharyngeal swabs had significantly greater bacterial richness than rectal swabs (Wilcoxon test, *P* < 0.001). Oropharyngeal samples from dogs exhibited higher bacterial richness than those of cats (*P* < 0.001), while cat rectal samples showed higher viral richness compared to dogs (*P* = 0.005). With respect to health status, bacterial richness was higher in oropharyngeal samples from healthy compared to sick dogs (*P* = 0.001). Conversely, viral richness was significantly higher in oropharyngeal swabs of diseased animals (cat oropharyngeal swabs, *P* = 0.034; dog oropharyngeal swabs, *P* = 0.013) and in rectal swabs of dogs (*P* < 0.001). Average viral abundance levels were also significantly higher in diseased animals (*P* < 0.05). Bacterial and viral community composition (beta diversity) differed significantly among sampling sites for both bacteria (PERMANOVA test, *R*^2^ = 0.26, *P* < 0.001) and viruses (*R*^2^ = 0.038, *P* < 0.001). For bacteria, the composition differed between cats and dogs in both swabs (*P* < 0.001), and between health status in oropharyngeal swabs (*P* = 0.005). Viral composition was significantly different between species (rectal swabs, *P* = 0.003; oropharyngeal swabs, *P* < 0.001) and health status (rectal swabs, *P* = 0.002; oropharyngeal swabs, *P* = 0.009) in both swabs.

### Possible disease associations

**Fig 4:**
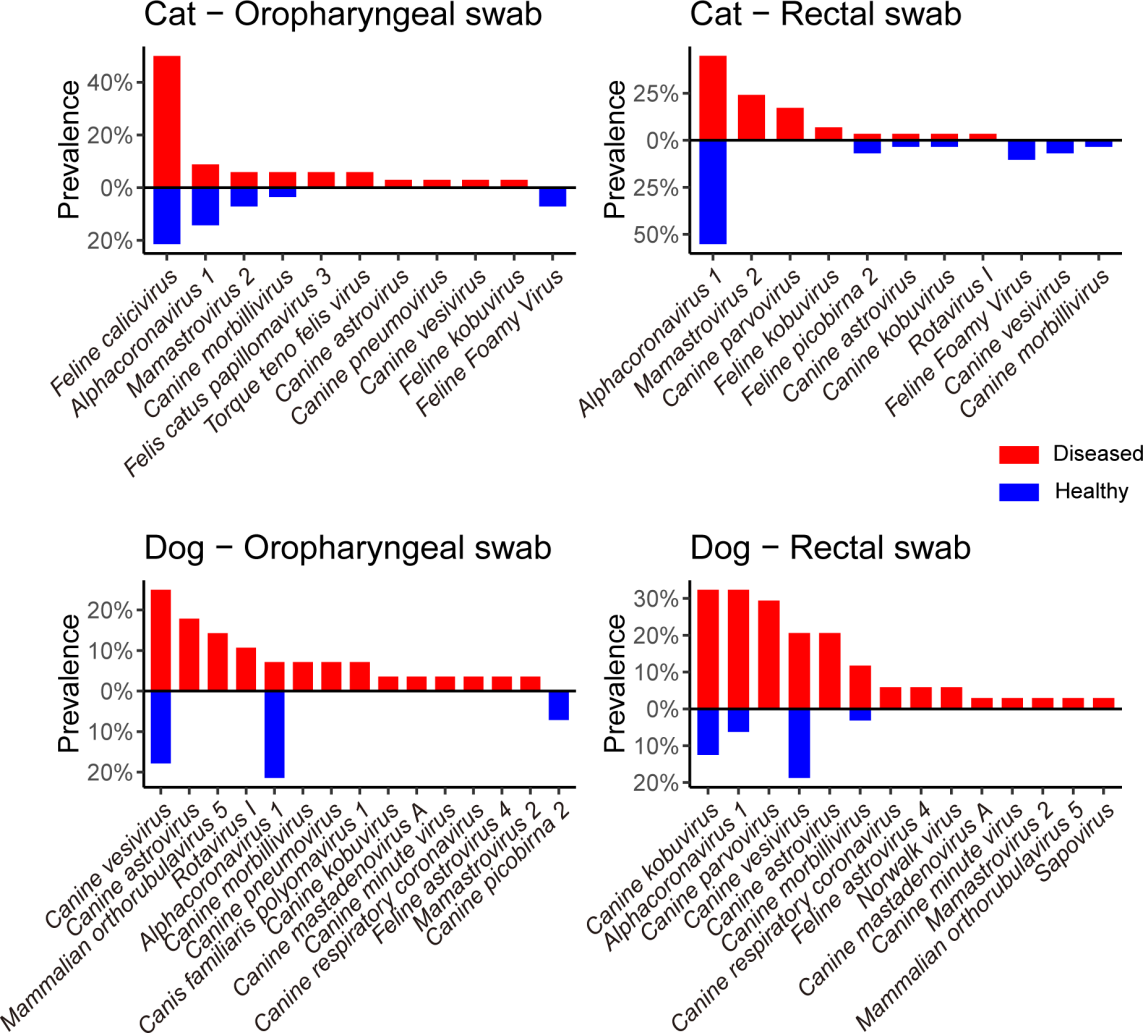
Differences in prevalence in viral species among healthy and diseased animals. The prevalence of viral species detected in specific samples, sorted by prevalence in diseased animals (red) and then by prevalence in healthy animals (blue).

Viral species potentially associated with respiratory and/or gastrointestinal diseases were inferred by comparing their prevalence among healthy and diseased animals showing either respiratory or gastrointestinal signs (Fig. 4). Of note, within the diseased group rectal swabs were solely collected from animals with gastrointestinal signs, while oropharyngeal swabs were solely obtained from those with respiratory symptoms. Thus, comparisons between sampling sites in diseased animals also imply a comparison between animals showing respiratory and gastrointestinal signs. Many viruses were only associated with diseased animals, such as *canine parvovirus*, *canine astroviruses*, and *mammalian orthorubulavirus 5*, and also showed specificity to particular sampling sites or animal species. All other viruses were detected in both healthy and diseased animals, although some were at greater prevalence levels in diseased than healthy animals, such as *feline calicivirus* and *canine morbillivirus* in dog samples. Interestingly, in the case of *alphacoronavirus 1*, disease associations might differ between sampling sites and animals. Indeed, this virus was only associated with disease in dog rectal samples (Fig. 4).

**Fig 5:**
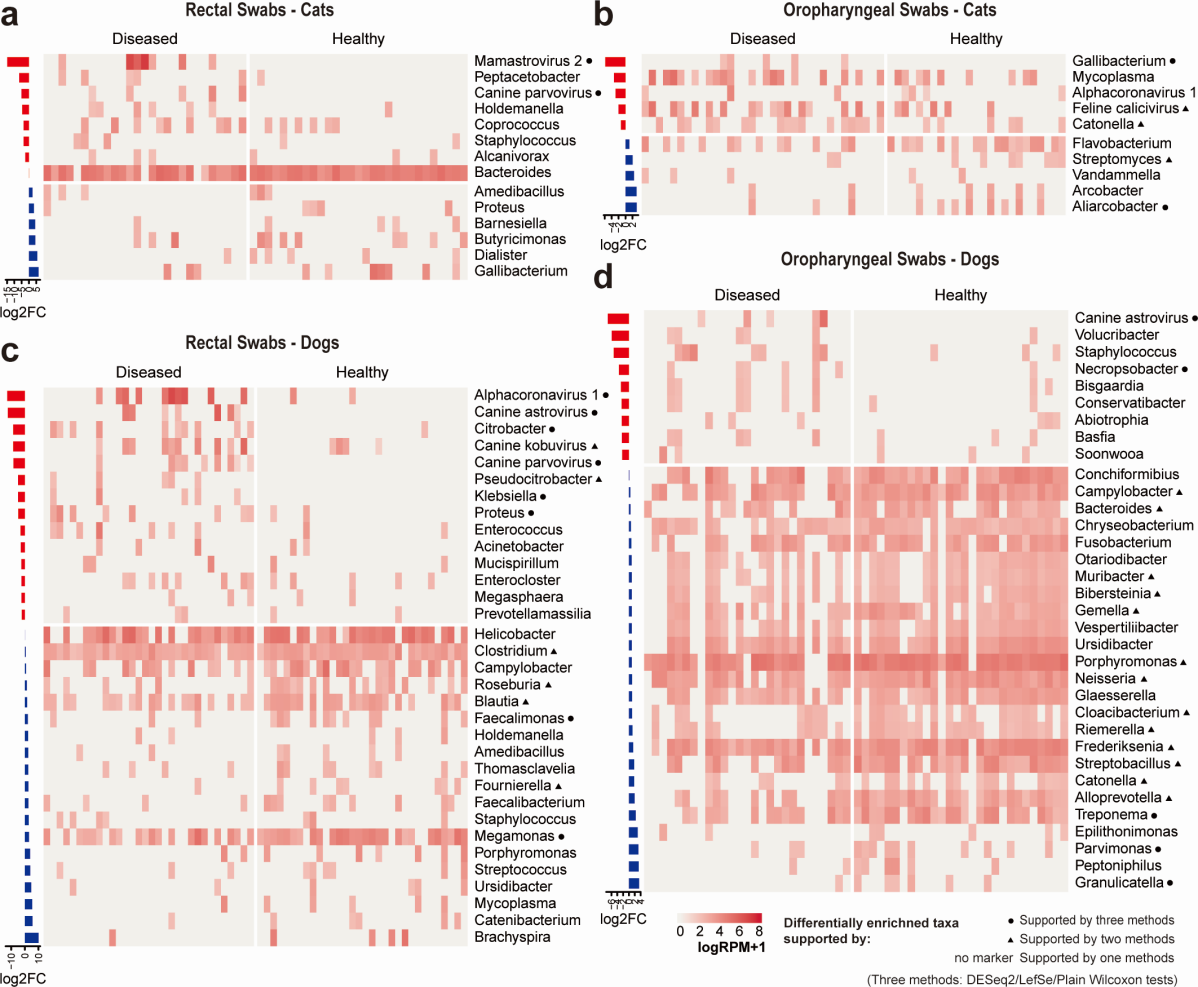
Differential abundance analysis (DAA) of the infectomes of healthy and diseased animals. (a−d) Heatmaps demonstrating the abundance differences of microbial taxa (bacterial and fungal genera; viral species) among diseased and healthy animals. DAAs were performed using three methods—LEfSe, DESeq2, and Wilcoxon tests. The bar plots on the left exhibit the log-transformed fold change in abundance (RPM) of the corresponding taxa.

Because potential pathogens may show significantly greater abundance in diseased animals, we performed differential abundance analysis (DAA) between healthy and diseased animals using three independent tests—DESeq2, LefSe, and Wilcoxon tests. Only microbial species/genera with more than five positive samples were included in this analysis. This analysis showed that six virus species and 26 bacterial genera were associated with disease (Fig. 5; Fig. S6 and S7). In contrast, 51 bacterial genera showed the opposite trend: higher abundance in healthy than diseased (Fig. 5; Fig. S6 and S7).

**Fig 6:**
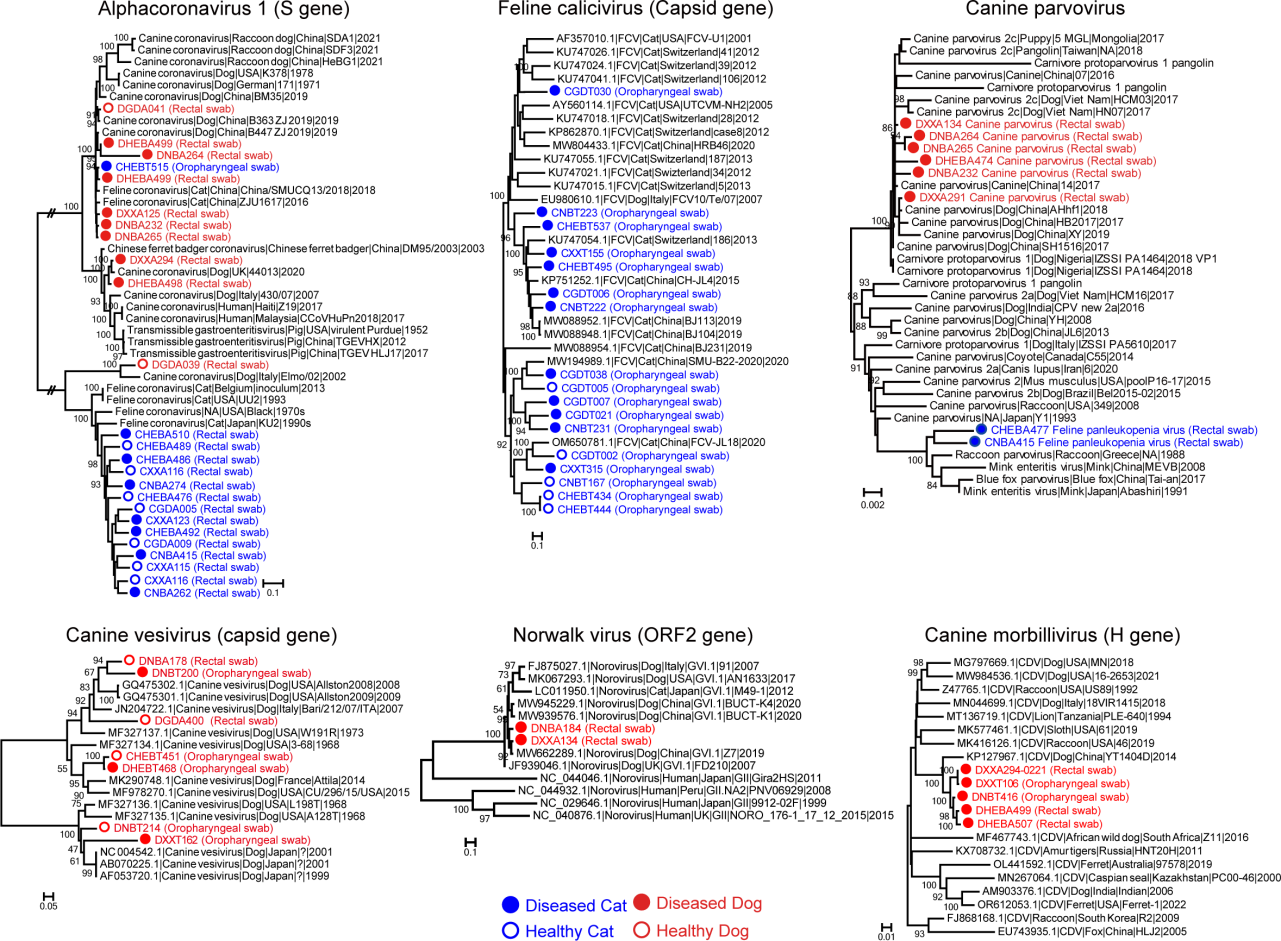
Maximum likelihood phylogenetic trees of six viral pathogens. The phylogenies were inferred using key functional genes (nucleotide sequences): Alphacoronavirus 1 (S gene), Feline calicivirus (capsid gene), Carnivore protoparvovirus 1 (VP1 gene), Nowalkvirus (ORF2 gene), Canine vesivirus (capsid gene), and Canine distemper virus (H gene). Host species and tissue type are indicated. The trees are midpoint rooted for clarity, with branch lengths reflecting the number of substitutions per site.

Differences in pathogenic potential and tissue/host preference were also apparent at the genotype/lineage level as shown in intra-specific phylogenetic trees of several potentially pathogenic and non-pathogenic virus species (Fig. 6). For *alphacoronavirus 1*, sequences from the canines-associated lineages were primarily found in sick dogs, whereas those from the feline-associated lineage were found in both healthy and diseased cats. Similarly, distinct lineages were associated with differences in pathogenicity for feline calicivirus, with certain lineages predominantly associated with diseased cats, while another lineage was associated with both healthy and diseased cats, suggesting opportunistic infections. In contrast, no such patterns were observed for *canine vesivirus* (Fig. 6).

### Shared infectious agents and zoonotic risks

**Fig 7:**
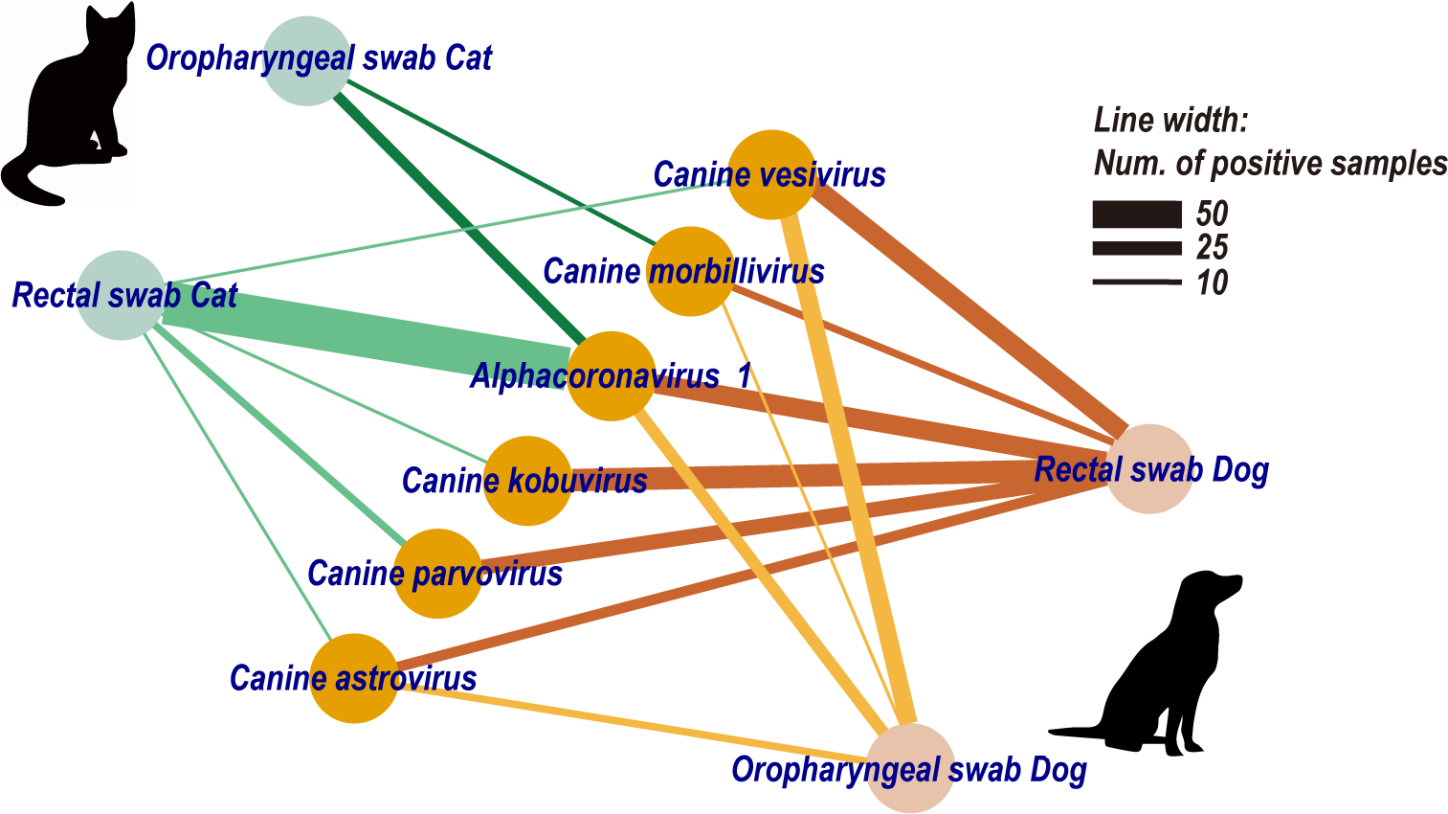
The virus-sharing network between cats and dogs. Nodes represent either viral species or specific sample types (rectal/throat swabs of cats/dogs). Lines between nodes indicate the presence of a specific viral species in corresponding samples, and the line width is proportional to the number of positive samples.

We identified six viral species that infected both cats and dogs, suggesting a history of cross-species transmission (Fig. 7). All six virus species were detected in rectal swabs of dogs and cats, with the exception that *canine morbillivirus* was not found in cat rectal swab. Four viral species (*canine morbillivirus and alphacoronavirus 1*, *canine vesivirus and canine astrovirus*) were additionally detected in oropharyngeal swabs. Of note, *alphacoronavirus 1* appeared at high prevalence in both species and sampling sites, suggesting a broad host range and tissue tropism (Fig. 6). Additionally, these data revealed a number of mixed infections (Fig. S8; Table S3). For instance, *alphacoronavirus 1* frequently co-occurred with *canine parvovirus* and *canine kobuvirus* (Chi-squared tests, *P* < 0.05), and significant positive correlations among their abundance were also detected (Spearman tests, *P* < 0.05).

Notably, we identified 27 known (opportunistic) zoonotic pathogens to humans, comprising 2 viral species, 23 bacterial species, and 2 fungal species (refer to Table S2). Of these, seven species were significantly more prevalent in rectal swabs (Chi-squared test, *P* < 0.05), whereas another eight species were more prevalent in oropharyngeal swabs. Many of these zoonotic pathogens (63%, 17 species) were detectable in both dogs and cats, with no statistically significant differences in their prevalence between the two host species (Chi-squared tests, *P* < 0.05). A similar pattern was observed across health status, where 62% of these pathogens (17 species) were detectable in both healthy and diseased animals.

## DISCUSSION

The infectomes of companion animals such as cats and dogs may have important implications for animal and perhaps human health (23). While previous studies have characterized infectomes through the metagenomic sequencing of total DNA [e.g., references (19, 24)], our study employed a meta-transcriptomic approach to specifically profile the actively transcribing portion of the infectome. This enabled comprehensive characterization of viruses as well as bacteria and fungi.

Our analysis revealed that bacteria dominated the healthy pet infectome, comprising an average 11.3% of total non-rRNA reads. The predominant bacterial genera identified, such as *Fusobacterium*, *Bacteroides*, *Phocaeicola*, and *Helicobacter* in rectal swabs, and *Porphyromonas*, *Capnocytophaga*, and *Bergeyella* in respiratory swabs, were largely consistent with previous studies (25–28). This implies that overall transcriptional activity correlates with genome abundance for dominant bacteria. Also, in accord with previous studies, our results showed that the microbiome of healthy animals differs between tissue types (25, 29), but less so between different host species (25). Some specific bacteria species/genera can be detected in both cats and dogs, and there is evidence suggesting that some of these may be zoonotic, causing diseases from diarrhea [*C. difficile* (30), *F. varium* (31)]*,* to serious bite-wound infections [*Pasteurella* spp. (32), *Capnocytophaga* spp. (33), and *Neissieria* spp. (34)].

Although fungi are considered part of the normal mycobiota in animals (35), metagenomic studies suggest that their abundance is much lower than bacteria, and they were rarely detected in this study. For instance, in a study on dog intestines, fungi comprised only 1% of the microbial DNA reads, while bacteria accounted for 98% (36). This is consistent with the notion that bacteria dominate the microbiome. Given the low abundance of most fungi, the actively transcribing portion might be even lower. While meta-transcriptomics is still capable of detecting abundant, actively transcribing fungi, which could indicate ongoing infections (e.g., candidiasis), it is important to note that the RNA extraction methods employed in this study did not involve fungal cell disruption techniques such as bead-beating, which are essential for enriching fungal DNA in metagenomics studies (37).

For the viral component of infectome, we focused specifically on mammal-associated viruses with likely direct relevance to animal and human health. RNA viruses dominated this component in terms of both diversity and abundance. In contrast to bacteriome which is dominated by commensal species, the pet virome consists of many well-known pathogens, such as *feline calicivirus* and *canine parvovirus*, as well as emerging viral pathogens like *canine vesivirus* (38), *canine kobuvirus* (39). While viruses exhibited lower overall abundance than bacteria, viral reads exceeded 10% of the total non-rRNA reads in some samples, indicating that the virome represents an important part of the active infectome. Notably, viral composition differed less between sampling sites (rectal or oropharyngeal) than bacteria, likely the result of systemic infection (which is the case for *canine morbillivirus* (40), etc.). Furthermore, our results documented the sharing of several viral species between cats and dogs, including viral species that pose potential zoonotic risks to humans such as *alphacoronavirus 1*, highlighting the public health importance of monitoring pets using meta-transcriptomics.

Comparative analysis between healthy and diseased animals provided further evidence on the potential pathogenic roles of several viruses with previously ambiguous disease associations, such as *canine astrovirus* (20). In particular, their enrichment in diseased compared to healthy animals suggests that they may play a role as opportunistic pathogens. Other viruses, including *canine vesivirus*, showed high prevalence regardless of health status, suggesting that they are normal components of the virome that may become pathogenic in contexts of disrupted homeostasis. While differential abundance testing identified taxa enriched in disease, limited sample sizes and genus-level resolution (bacteria and fungi) could have obscured the detection of some pathogens.

Importantly, we detected a number of zoonotic viral, bacterial, and fungal pathogens that might cause opportunistic infections in humans, including alphacoronavirus 1 (41), Norwalk virus (42), *C. difficile* (30), *C. jejuni* (43), and *C. albicans* (44). Their presence in these companion animals highlights potential risks of disease transmission to humans, especially immunocompromised individuals or those with frequent animal contact. Comprehensive surveillance of pets and their owners is therefore warranted to better understand zoonotic risks. Importantly, our results revealed that while these potential zoonotic pathogens exhibited distinct sampling site preferences, 22% (six species) were detectable in both sampling sites. Strikingly, 62% of these zoonotic pathogens were detectable in both healthy/diseased dogs and cats. These findings underscore the importance of surveillance in both dogs and cats, regardless of their health status, while targeting specific sampling sites for detecting intended pathogens.

Interestingly, our analysis identified fewer zoonotic viral species (2 species) than zoonotic bacterial species (23 species) in companion animals. This contrasts with studies of other domestic animals such as pigs, where numerous zoonotic viruses have been characterized. For example, metagenomic analyses of pigs (15) have revealed the presence of zoonotic viruses like influenza A virus, hepatitis E virus, and multiple arbovirus species (e.g., Japanese encephalitis virus, Getah virus, and Zika virus) in addition to common zoonotic bacterial pathogens such as *Streptococcus suis*. The relatively lower viral zoonotic risk observed here for companion animals may be attributable to differences in viral host range, tropism, and transmission dynamics between companion animals versus livestock species. Nonetheless, the zoonotic viral threats identified again highlight the importance of viral surveillance even in companion animal populations.

The findings of our study could be further strengthened by addressing several limitations. First, while we used meta-transcriptomics to characterize the total infectome, this approach may miss some microorganisms that are not actively transcribing. Second, several potential factors influencing infectome composition, such as animal feed, medication, or household hygiene, were not collected, and these unmeasured variables may confound our results. Third, the study focused solely on samples from Chinese domestic animals, so it is unclear whether our findings are applicable to other regions or countries with different environmental and socio-economic conditions. These limitations highlight the need for further research that incorporates a broader range of infectome-driving factors and geographical contexts to fully understand the infectome dynamics of companion animals.

## MATERIALS AND METHODS

### Sample collection

Between March 2021 and March 2022, we sampled domestic animals (i.e., cats and dogs) from pet hospitals and pet care centers located in five major cities distributed throughout China: Guangzhou (Southern China), Shenzhen (Southern), Xinxiang (Central), Ningbo (Eastern), and Harbin (Northern). Both healthy animals and diseased animals were enrolled. The diseased animals had either respiratory (e.g., sneezing, coughing, nasal discharge) or gastrointestinal symptoms (e.g., diarrhea, vomiting, and loss of appetite) after the initial diagnosis by the veterinarians who carried out the sampling. Healthy animals showed no clinical symptoms at the time of sampling and were taken to the facility for health check, cleaning, and beauty services. For each animal, oropharyngeal or rectal swab sampling was performed depending on the clinical symptoms. The samples were immediately immersed in virus preservation solution (SHENQI Biotech), placed on dry ice, and then transferred to −80°C refrigerator for storage. Detailed sample information is provided in Table S1.

### Sample processing and meta-transcriptomics analyses

Total RNA was extracted from 239 individual swab samples using the RNeasy Plus universal mini kit (QIAGEN) according to the manufacturer’s instructions. The concentration of extracted RNA was determined using a Qubit RNA High Sensitivity Kit (Thermo Fisher Scientific). RNA sequencing library construction was performed using the Trio RNA-Seq Library Preparation Kit (NuGEN Technologies) which targeted low-concentration RNA as starting material and used an AnyDeplete probe (NuGEN Technologies) to remove host ribosomal RNA. The concentration and quality of constructed library were determined using Qubit dsDNA Quantification Assay Kit (Thermo Fisher Scientific) and Qsep100 (Bioptic), respectively. Paired-end (150 bp) sequencing of the libraries was performed on the Illumina NovaSeq platform. The quality control of subsequent sequencing reads was performed using BBDuk (version 38.62) (downloaded from https://jgi.doe.gov/data-and-tools/software-tools/bbtools/). rRNA reads were removed by mapping against a comprehensive rRNA reference sequence collection downloaded from the SILVA database (45) (https://www.arb-silva.de/) using bowtie2 (version 2.3.5.1) (46). The deduplication of the reads was performed using CD-HIT (version 4.8.1) (47), and the processed reads were then *de novo* assembled into contigs using MEGAHIT (version 1.2.8) (48) for subsequent microbial identification and profiling.

### Profiling vertebrate viruses

We identified potential RNA and DNA viruses by comparing the assembled contigs against an NCBI reference virus database using blastn (49) (version 2.9.0) and an NCBI non-redundant protein database (nr) using Diamond blastx (50) (version 0.9.25). Contigs with at least one significant hit (*E* value <10^−5^) to known viral sequences in either database were collected. The resulting viral contigs were subsequently compared to the non-redundant nucleotide database (nt) to identify and remove contigs or regions in the contigs that were related to host or bacterial genomes [significant alignments (*E* value <10^−5^) to domain Bacteria or class Chordata sequences in the nt database]. The remaining viral contigs were categorized to the species level based on sequence homology with reference viral genomes as well as that with each other. Novel viral species were defined based on the guidelines provided by the International Committee on Taxonomy of Viruses.

Among all the viral species identified, we only characterized the likely mammal-associated virome, with the component viruses determined based on whether they fell within well-defined vertebrate-specific viral genera or families following phylogenetic analysis (51). Specifically, we reconstructed the phylogenetic tree of all detected viruses with conserved polymerase proteins, using a manually curated tree backbone from the nr database (accession codes were provided in the supplementary materials). Viruses were assigned genera and family annotations based on their placement within the monophyletic clades of specific genera or families. The quantification of each viral species was carried out by mapping non-rRNA reads to the viral genomes identified in this study as well as to related reference sequences. Read counts mapped to each sequence were summed at the species level. Index-hopping filtering was performed on the read count matrix using a threshold of 0.1%. The filtered read count matrix was then normalized by total non-rRNA read count to generate an RPM table.

### Profiling the composition of bacteria and fungi

We identified bacterial and fungal genera and quantified their abundance by mapping non-rRNA reads to a set of reference genomes. The reference genomes of bacteria were retrieved from the Genome Taxonomy Database (52) (GTDB) release 214.1, which is a widely used database of curated bacteria genomes and taxonomy. For fungi, we used all representative fungal genomes from the NCBI Genome Assembly database. Before mapping reads to genomes, rRNA regions in these genomes were masked with the ambiguous nucleotide code “N” to reduce false-positive alignments to the highly conserved rRNA gene. This was done by performing a blastn (49) search against SILVA rRNA database (45) (release 138) to locate rRNA and then mask them with a custom python script. When the reference genomes were prepared, non-rRNA reads were aligned against these genomes using bowtie2 (46). The sensitivity of the read alignment process was set to “—very-fast.” To reduce false-positives, we scanned the consensus sequences from read alignments for a set of universal marker genes using BUSCO v5.5.0 (53). If none of the marker genes were present in a consensus sequence, the whole alignment was considered as false-positive and removed. After these steps, read counts mapped to each genome were summed by genus, and index-hopping filtering was performed on the genera-level read count matrix as described above. The filtered read count matrix was then normalized by the total non-rRNA read count to generate an RPM table. Bacterial and fungal genera at low abundance (RPM <100) were removed.

### Identification of potential zoonotic pathogens

To identify potential zoonotic pathogens, we conducted species-level profiling for several bacterial and fungal genera known to harbor human pathogens (viruses were already identified to the species level; see above). A list of these genera was obtained from the KEGG DISEASE database (54). For bacteria, we extracted two phylogenetically informative genes—rpoB (55) and gyrB (36)—from the consensus sequences of these genera generated during read mapping (as described above). For fungi, we used ACT1 and TEF1 genes instead, which are also widely used in phylogenetic analyses of fungi (56). The nucleotide sequences of these genes were aligned with reference sequences (reference sequences of bacteria were retrieved from GTDB, and fungi sequences were retrieved from UFCG) of specific genera using MAFFT (57). Phylogenetic trees were then estimated using the maximum likelihood method in IQ-TREE (58) (employing the GTR + I + F+G_4_ substitution model). Clustering of the taxa generated here to known species was visually inspected on the phylogenies. Additionally, we calculated the average nucleotide identity (ANI) between the mapped consensus sequence and reference genomes. The identification of zoonotic pathogens to species resolution was supported by both ANI >97% and evidence from marker genes phylogeny.

### Diversity of the pet infectome

We assessed the alpha diversity of the pet infectome by examining the richness of bacterial genera and viral species richness per sample. Due to low fungal diversity in our samples, these were omitted from the analyses of both alpha and beta diversity. We employed Wilcoxon rank-sum tests to compare the mean richness per sample across different sampling sites (oropharyngeal swabs vs rectal swabs), species (cats vs dogs), and health status (healthy vs diseased). Statistical significance was determined at a threshold of *P* < 0.05. Notably, in the comparisons between sampling sites and species, only samples from healthy animals (*n* = 117) were included.

Subsequently, we visualized the beta diversity of the pet infectome using ordination plots depicting microbial community compositions. For bacteria, we utilized Bray-Curtis distance metrics for beta diversity characterization and principal coordinate analysis for visualization. For viruses, Bray-Curtis distance was similarly utilized, but visualization was performed using t-distributed stochastic neighbor embedding, as the viral abundance matrix (viral species-by-sample) exhibited significant sparsity. To assess differences in pet microbial composition across sampling sites, species, and health status, we conducted PERMANOVA using the adonis2 function within the R package vegan. Statistical significance was determined based on 999 permutations to generate *P* values.

### Differential Abundance Analyses

To identify microbial taxa potentially associated with diseases in dogs and cats, we utilized DAAs. We performed three independent analyses using different methods of DAA—DESeq2 (59), LefSe (60), and Wilcoxon tests—to produce as robust results as possible. These analyses were performed separately for each type of sample (i.e., dog rectal swab, dog oropharyngeal swab, cat rectal swab, and cat oropharyngeal swab). Only those bacterial/fungal genera or viral species with more than five positive samples were included in the analyses.

For the DESeq2 analysis, we used the abundance matrix (RPM values, microbial taxa-by-sample) as input. Significant microbial taxa were identified based on an adjusted *P* value of <0.05 and a log2FC (log-transformed fold change in read count between groups) exceeding 1 or below −1. For LefSe analysis, the abundance matrix (RPM values) was used as input. Results with LDA scores >2 and *P* values <0.01 were deemed significant (as recommended by the software). We performed Wilcoxon tests on each microbial taxon to compare their abundance (log-transformed RPM values) between disease and healthy pets. Any result with a *P* value <0.05 was deemed significant. Differential abundance analyses may produce different results, and those taxa supported by more than one method are thought to be more robust (61). Finally, the differentially enriched taxa identified by the above methods were visualized by a heatmap.

### Phylogenetic analyses

We estimated the evolutionary history of six (potential) viral pathogens: *alphacoronavirus 1*, *feline calicivirus*, *canine parvovirus*, *Norwalk virus*, *canine vesivirus*, and *canine morbillivirus*. Accordingly, phylogenetic trees were inferred based on key functional genes of each virus: the S gene for *alphacoronavirus 1*, capsid gene for *feline calicivirus*, VP1 gene for *canine parvovirus,* ORF2 gene for *Norwalk virus*, capsid gene for *canine vesivirus* and H gene for *canine morbillivirus*. We used IQ-TREE (58) (version 2.2.0.3) to estimate phylogenies assuming a GTR + I + G_4_ model of nucleotide substitution. Bootstrap resampling (1,000 replications) using ultra-fast bootstrapping option (−bb 1,000) was used to assess the robustness of individual nodes.

We similarly performed phylogenetic analysis on bacteria. To retrieve marker gene sequence of bacteria, we aligned reads to representative genomes of several candidate genera (genera which may contain zoonotic or pathogenic species) using bowtie2 (version 2.5.0) (46) and extracted rpoB gene (RNA polymerase beta subunit) from the consensus sequences. We then aligned these extracted nucleotide sequences together with reference sequences from GTDB release 214.1 (52) using MAFFT v7.515 (57) (L-INS-i algorithm, max iteration set to 1,000). Phylogenetic trees were estimated using IQ-TREE (version 2.2.0.3) (58), again employing the GTR + I + G_4_ substitution model.

## SUPPLEMENTAL MATERIAL

Supplemental figures msphere.00439-24-s0001.docx Figures S1 to S8.

Table S1 msphere.00439-24-s0002.xlsx Sample information.

Table S2 msphere.00439-24-s0003.xlsx Zoonotic pathogen manifest.

Table S3 msphere.00439-24-s0004.xlsx Virus coinfection statistics.

ASM does not own the copyrights to Supplemental Material that may be linked to, or accessed through, an article. The authors have granted ASM a non-exclusive, world-wide license to publish the Supplemental Material files. Please contact the corresponding author directly for reuse.
