# Supplementary material for: Meta-transcriptomic analysis of companion animal infectomes reveals their diversity and potential roles in animal and human disease
Source: mSphere. 2024 Jul 16;9(8):e00439-24. doi: 10.1128/msphere.00439-24 (PMC11351045; doi:10.1128/msphere.00439-24)
Supplement: Supplemental figures — Figures S1 to S8. [file msphere.00439-24-s0001.docx]

**Supplementary Information for**

“Meta-transcriptomic analysis of companion animal infectomes reveals their diversity and potential roles in animal and human disease”

**Supplementary Figures**


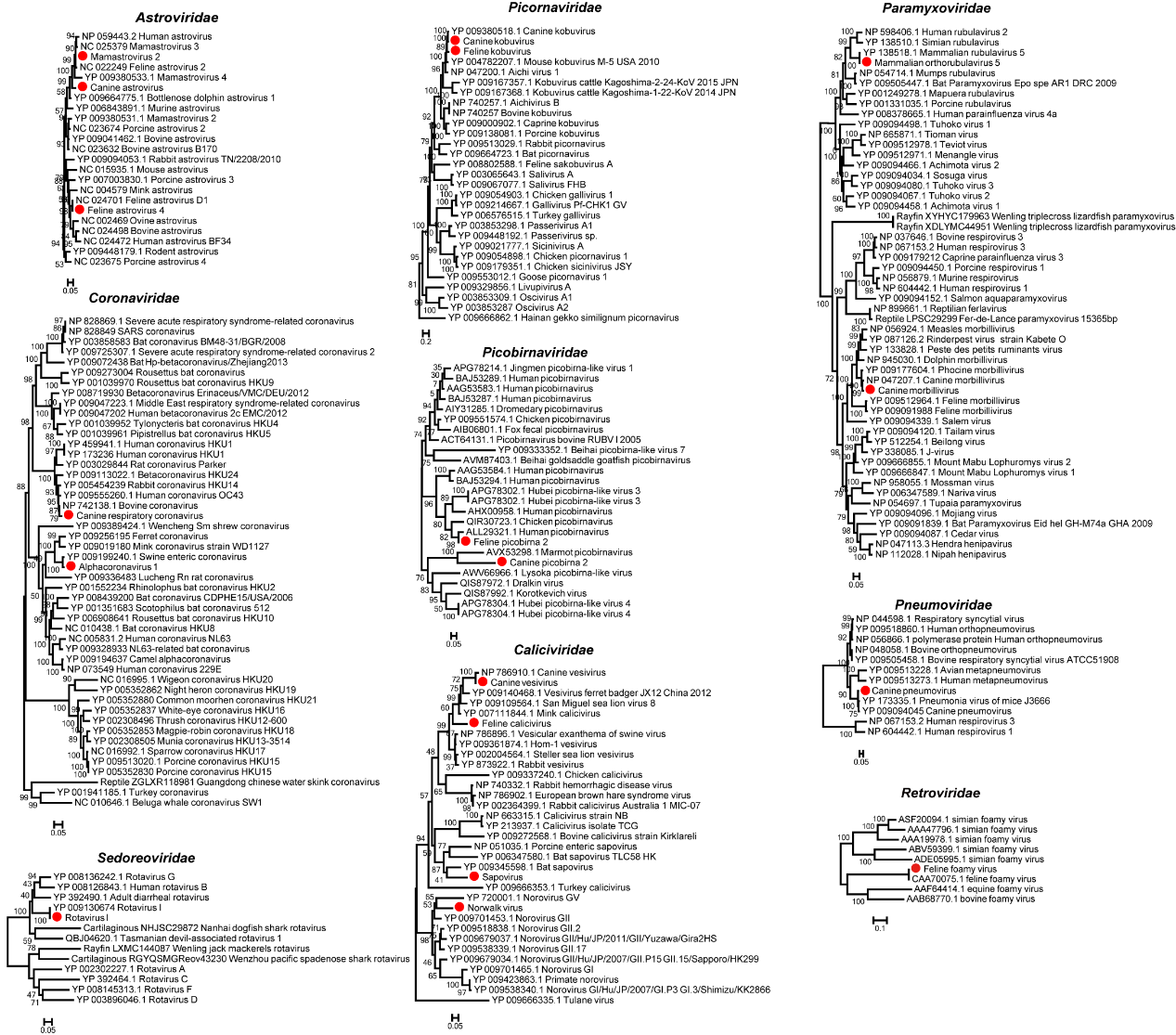


**Figure S1. Maximum likelihood phylogenetic trees of RNA viruses.** The phylogenies were inferred based on multiple sequence alignments of the RNA-dependent RNA polymerase (RdRp) protein, with the exception of the *Retroviridae* for which the reverse transcriptase (RT) protein was used The trees are midpoint rooted for clarity, with branch lengths reflecting the number of substitutions per site.


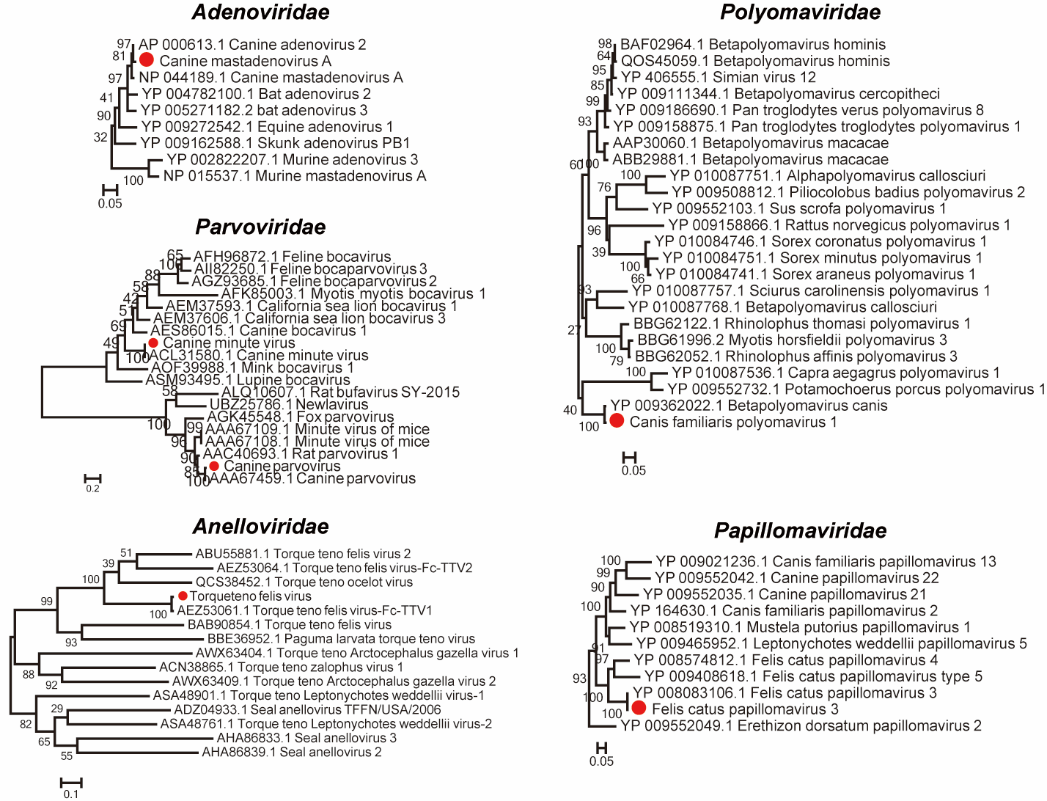


**Figure S2. Maximum likelihood phylogenetic trees of DNA viruses.** The phylogenies were inferred using DNA polymerase protein (*Adenoviridae*), NS1 protein (*Parvoviridae*), ORF1 protein (*Anelloviridae*), E1 protein (*Papillomaviridae*), and LT-Ag protein (*Polyomaviridae*). The trees are midpoint rooted for clarity, with branch lengths reflecting the number of substitutions per site.


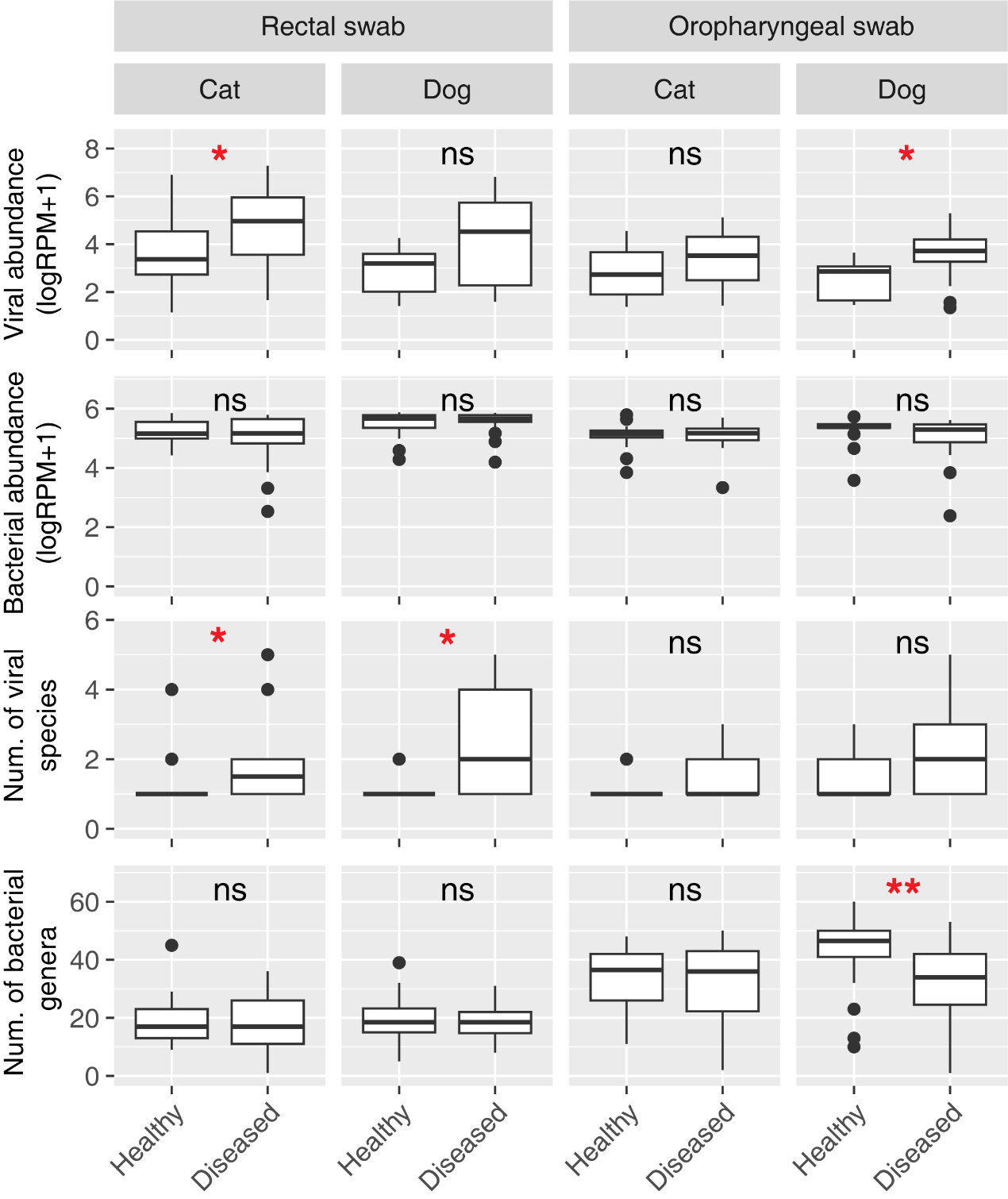


**Figure S3. Comparisons of viral and bacterial abundance and diversity in healthy and diseased animals.** Statistical significance was determined using two-sided Wilcoxon rank-sum tests. ns: not significant (p > 0.05), *: 0.01 < p ≤ 0.05, **: 0.001 < p ≤ 0.01.


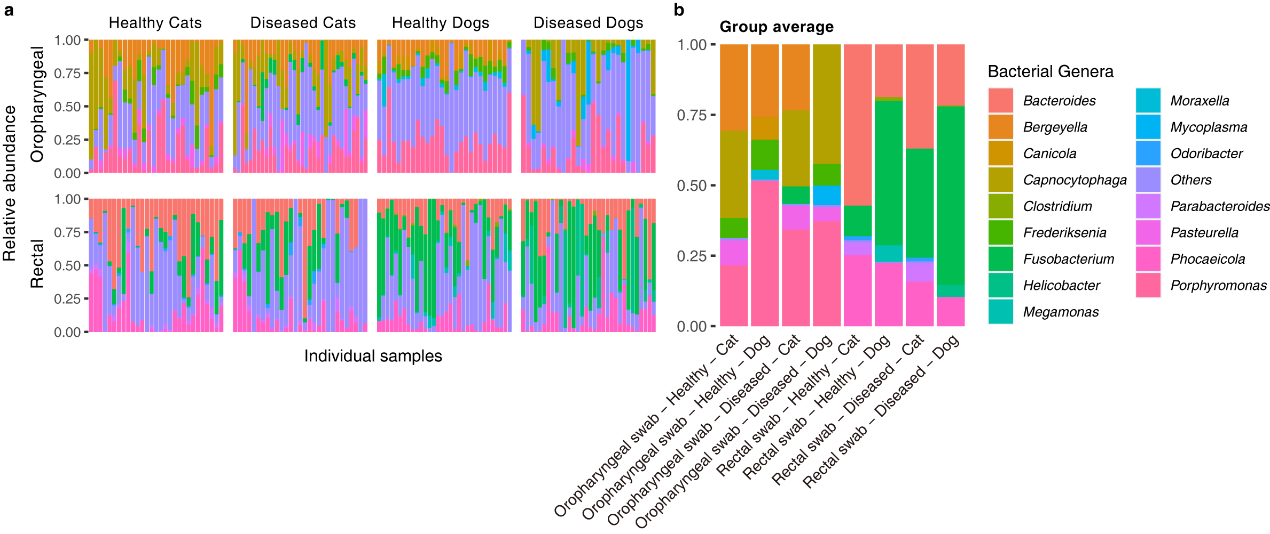


**Figure S4. Bacterial community composition in healthy and diseased cats and dogs.** (a) Bacterial genera composition in each sample. (b) Bar plot showing the average relative abundances of major bacterial genera within each group. The y-axis represents the relative abundance, and the x-axis labels denote the different groups. Colors are used to distinguish different bacterial genera (including top 5 most abundant genera in of group).


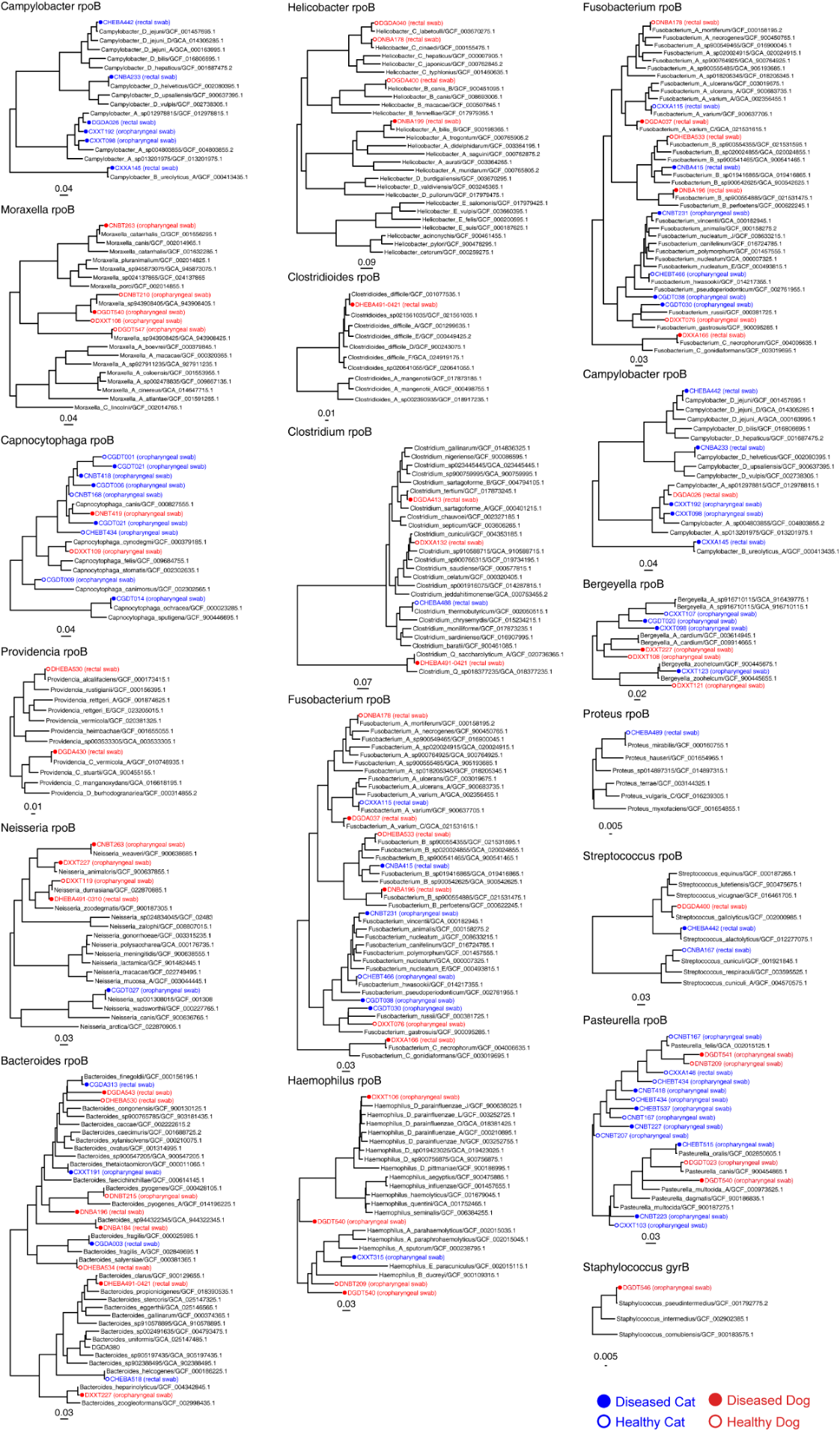


**Figure S5. Maximum likelihood phylogenetic trees of potential zoonotic bacterial species.** The phylogenies were inferred using nucleotide sequences of rpoB gene. The trees are midpoint rooted for clarity, with branch lengths reflecting the number of substitutions per site.

**
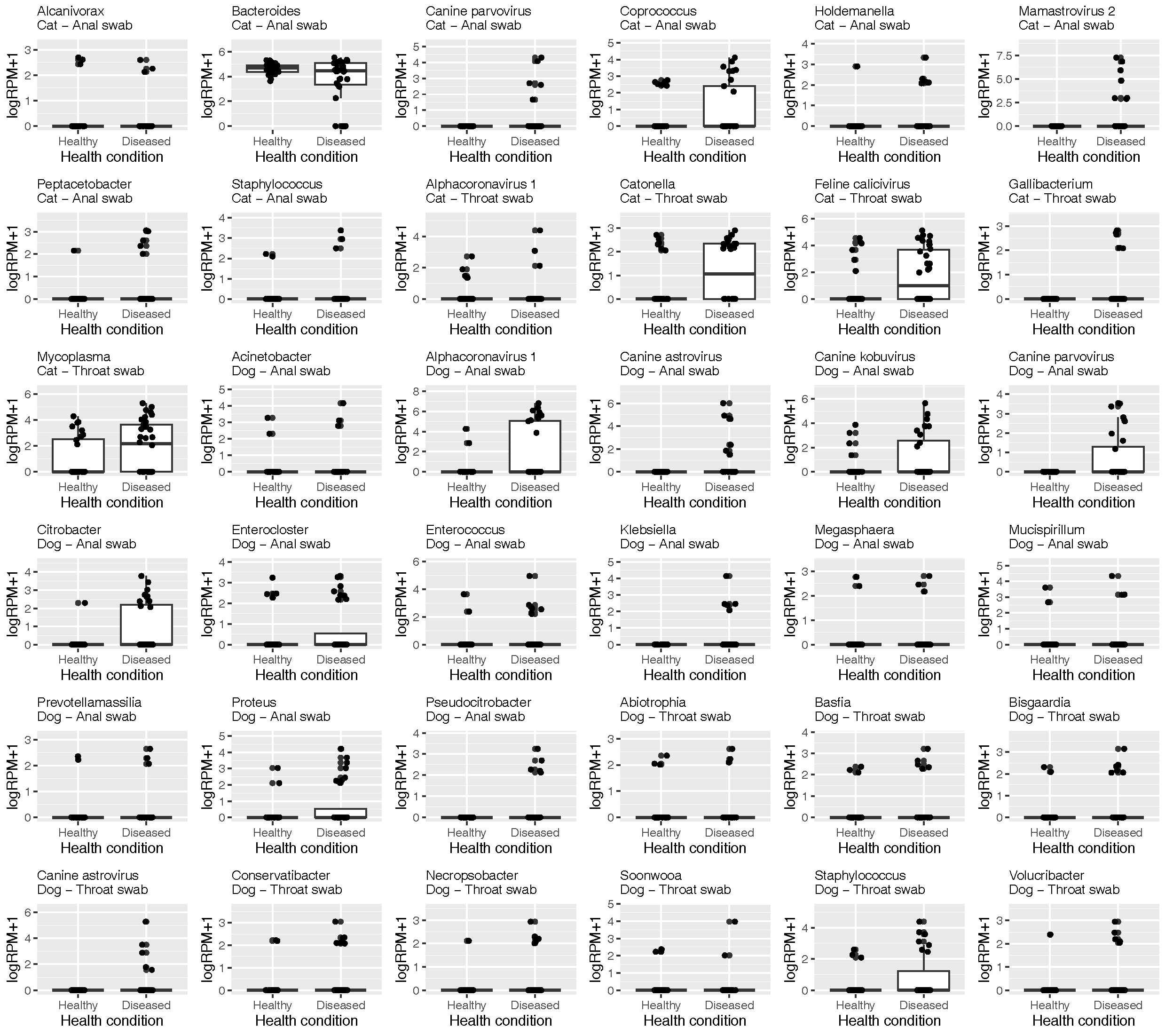
**

**Figure S6. Bacterial genera and Viral species enriched in diseased animals.** These genera were supported at least by one of the three DAA methods (DESeq2, LefSe and Wilcoxon’s tests).





**Figure S7. Bacterial genera enriched in healthy animals.** These genera were supported at least by one of the three DAA methods (DESeq2, LefSe and Wilcoxon’s tests).


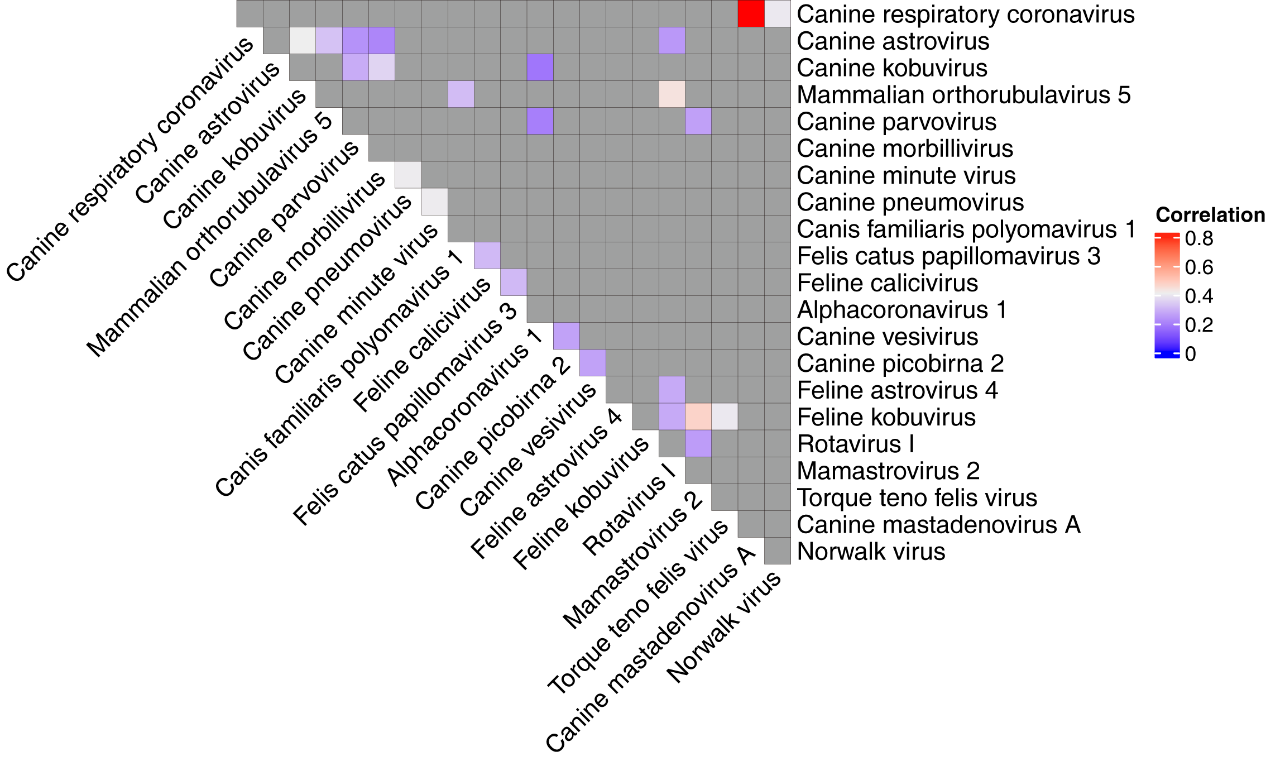


**Figure S8. Heatmap illustrating virus co-infections in companion animals.** Colors represent the Spearman correlation between the abundance level of two viruses. All the significant correlations (p < 0.05) tested here are positive, with the red color indicating a stronger positive correlation. The gray color indicates non-significant correlations.

**Supplementary Tables (see separate spreadsheet files)**

**Table S1.** Detailed Sample Information of Animals Sampled in This Study.

**Table S2.** Prevalence of Zoonotic Agents and Statistical Assessment of Host and Tissue Distribution.

**Table S3.** Co-infection Prevalence and Statistical Measures among Pairs of Viruses.
